# Supplementary material for: Superfluidity enhanced by spin-flip tunnelling in the presence of a magnetic field
Source: Sci Rep. 2016 Sep 16;6:33320. doi: 10.1038/srep33320 (PMC5025894; doi:10.1038/srep33320)
Supplement: Supplementary Information [file srep33320-s1.pdf]

Supplementary materials for 'Superfluidity enhanced by spin-flip tunnelling in the presence of a magnetic field'

Jun-Hui Zheng,[1] Daw-Wei Wang,[1,2] and Gediminas Juzeliūnas[3]

[1] *Department of Physics, National Tsing Hua University, Hsinchu, Taiwan*
[2] *Physics Division, National Center for Theoretical Sciences, Hsinchu, Taiwan*
[3] *Institute of Theoretical Physics and Astronomy, Vilnius University, A. Goštauto 12, Vilnius 01108, Lithuania*

I. DERIVATION OF D_φ

According to Eq.(10) of the main text, applying the BCS mean-field approximation, the interaction term takes the form

$$H_{\rm int} = \frac{2\Delta^2 A}{|g|} + \sum_{\mathbf{k}, j=\Uparrow,\Downarrow} \left(\Delta_j \psi^\dagger_{j\uparrow,\mathbf{k}} \psi^\dagger_{j\downarrow,-\mathbf{k}} + H.c. \right), \tag{1}$$

where $\Delta = |\Delta_\Uparrow| = |\Delta_\Downarrow|$, with $\Delta^*_\Uparrow = \Delta_\Downarrow = \Delta_{\rm R} + i\Delta_{\rm I}$. By using the Fermi communtation relations, the second term of Eq.(1), denoted as $V_{\rm int}$, can be represented in a symmetrised manner as

$$V_{\rm int} \equiv \frac{1}{2} \sum_{\mathbf{k}j} \left[\Delta_j (\psi^\dagger_{j\uparrow,\mathbf{k}} \psi^\dagger_{j\downarrow,-\mathbf{k}} - \psi^\dagger_{j\downarrow,\mathbf{k}} \psi^\dagger_{j\uparrow,-\mathbf{k}}) + H.c. \right] . \tag{2}$$

By defining a row field operator $\Psi^\dagger_\mathbf{k} = [\psi^\dagger_{\Uparrow\uparrow,\mathbf{k}}, \psi^\dagger_{\Uparrow\downarrow,\mathbf{k}}, \psi^\dagger_{\Downarrow\uparrow,\mathbf{k}}, \psi^\dagger_{\Downarrow\downarrow,\mathbf{k}}]$, we obtain a concise form

$$V_{\rm int} \equiv \frac{1}{2} \sum_\mathbf{k} \Psi^\dagger_\mathbf{k} \left(i\Delta_{\rm R} \tau_0 \otimes \sigma_y + \Delta_{\rm I} \tau_z \otimes \sigma_y \right) \Psi^{\dagger T}_{-\mathbf{k}} + H.c., \tag{3}$$

where ^{T} stands for a transposed matrix, so $\Psi^{\dagger T}_{-\mathbf{k}}$ is a column field operator. In a diagonal representation of the single particle problem, the field operators read

$$C_\mathbf{k} = V_\varphi \Psi_\mathbf{k} , \qquad C^\dagger_\mathbf{k} = \Psi^\dagger_\mathbf{k} V^\dagger_\varphi = \Psi^\dagger_\mathbf{k} V^{-1}_\varphi , \tag{4}$$

where $\Psi_\mathbf{k} \equiv \left(\Psi^\dagger_\mathbf{k}\right)^\dagger = [\psi_{\Uparrow\uparrow,\mathbf{k}}, \psi_{\Uparrow\downarrow,\mathbf{k}}, \psi_{\Downarrow\uparrow,\mathbf{k}}, \psi_{\Downarrow\downarrow,\mathbf{k}}]^T$ is a column matrix. Therefore the field operators entering Eq.(3) can be represented as in terms of the normal modes as

$$\Psi^\dagger_\mathbf{k} = C^\dagger_\mathbf{k} V_\varphi , \qquad \Psi^{\dagger T}_\mathbf{k} = V^T_\varphi C^{\dagger T}_\mathbf{k} , \tag{5}$$

giving

$$V_{\rm int} \equiv \frac{1}{2} \sum_\mathbf{k} \left(C^\dagger_\mathbf{k} D_\varphi \, C^{\dagger T}_{-\mathbf{k}} + C^T_{-\mathbf{k}} D^\dagger_\varphi \, C_\mathbf{k} \right) , \tag{6}$$

with

$$D_\varphi = V_\varphi \left(i\Delta_{\rm R} \tau_0 \otimes \sigma_y + \Delta_{\rm I} \tau_z \otimes \sigma_y \right) V^T_\varphi . \tag{7}$$

where we used the fact that the unitary transformation V_φ is independent of $\mathbf{k}$. The operator $V_{\rm int}$ can be rewritten in a matrix form as

$$V_{\rm int} = \frac{1}{2} \sum_\mathbf{k} [C^\dagger_\mathbf{k}, C^T_{-\mathbf{k}}] \begin{bmatrix} 0 & D_\varphi \\ D^\dagger_\varphi & 0 \end{bmatrix} \begin{bmatrix} C_\mathbf{k} \\ C^{\dagger T}_{-\mathbf{k}} \end{bmatrix}, \tag{8}$$

giving the interaction term featured in Eq.(11) of the main text.

Specifically for $\varphi=0$, we have $V_0=\exp[-i\frac{\pi}{4}\tau_y]$ and $V_0^T=\exp[i\frac{\pi}{4}\tau_y]$, which satisfies $V_0\tau_z V_0^T=\tau_x$, giving

$$D_0 = i\Delta_{\rm R}\tau_0\otimes\sigma_y + \Delta_{\rm I}\tau_x\otimes\sigma_y \,. \tag{9}$$

For another case $\varphi=\pi/2$, we have $V_{\pi/2}=\exp[-i\frac{\theta}{2}\tau_y\otimes\sigma_y]$ and $V_{\pi/2}^T=V_{\pi/2}$. Therefore

$$V_{\pi/2}\tau_0\otimes\sigma_y V_{\pi/2} = (\tau_0\otimes\sigma_y\cos\theta - i\tau_y\otimes\sigma_0\sin\theta) \,, \tag{10}$$

$$V_{\pi/2}\tau_z\otimes\sigma_y V_{\pi/2} = \tau_z\otimes\sigma_y, \tag{11}$$

giving

$$D_{\pi/2} = \Delta_{\rm R}\left(i\tau_0\otimes\sigma_y\cos\theta + \tau_y\otimes\sigma_0\sin\theta\right) + \Delta_{\rm I}\tau_z\otimes\sigma_y. \tag{12}$$

II. GROUND STATE ENERGY

Let us denote the eigenvalues of the Bogoliubov-DeGuinne term to be $E^{\varphi}_{\alpha,\mathbf{k}}\geq 0$ with $\alpha=1,2,3,4$ and their corresponding operators as $d_{\alpha\mathbf{k}}$. The Hamiltonian in this eigenbasis reads:

$$\begin{aligned} H &= \frac{1}{2}\sum_{\alpha,\mathbf{k}}\left\{E^{\varphi}_{\alpha,\mathbf{k}}d^{\dagger}_{\alpha,\mathbf{k}}d_{\alpha,\mathbf{k}} - E^{\varphi}_{\alpha,\mathbf{k}}d_{\alpha,-\mathbf{k}}d^{\dagger}_{\alpha,-\mathbf{k}}\right\} + 2\sum_{\mathbf{k}}\varepsilon_{\mathbf{k}} + \frac{2\Delta^2 A}{|g|} \\ &= \sum_{\alpha,\mathbf{k}}E^{\varphi}_{\alpha,\mathbf{k}}d^{\dagger}_{\alpha,\mathbf{k}}d_{\alpha,\mathbf{k}} - \frac{1}{2}\sum_{\alpha,\mathbf{k}}E^{\varphi}_{\alpha,\mathbf{k}} + 2\sum_{\mathbf{k}}\varepsilon_{\mathbf{k}} + \frac{2\Delta^2 A}{|g|}. \end{aligned} \tag{13}$$

At zero temperature, the ground state has a zero number of Bololiubov excitations $\left\langle d^{\dagger}_{\alpha,\mathbf{k}}d_{\alpha,\mathbf{k}}\right\rangle=0$ because all $E^{\varphi}_{\alpha,\mathbf{k}}\geq 0$. Thus the ground-state energy becomes

$$\mathcal{E} = \frac{2\Delta^2 A}{|g|} + \sum_{\mathbf{k}}\left(2\varepsilon_{\mathbf{k}} - \frac{1}{2}\sum_{\alpha}E^{\varphi}_{\alpha,\mathbf{k}}\right). \tag{14}$$

A. Ground energy for single layer limit

In the following, we give the calculation for the ground energy for the case $t=0$, so that $E^{\varphi}_{\alpha,\mathbf{k}}=|\sqrt{\left(\frac{k^2}{2}-\mu\right)^2+\Delta^2}\pm\Omega|$. Explicitly, the ground energy becomes

$$\mathcal{E} = 2\Delta^2\sum_{\mathbf{k}}\frac{1}{\mathbf{k}^2/m+\epsilon_b} + \sum_{\mathbf{k}}(\frac{\mathbf{k}^2}{m}-2\mu) - \sum_{\mathbf{k},\pm}\left|\sqrt{\left(\frac{\mathbf{k}^2}{2m}-\mu\right)^2+\Delta^2}\pm\Omega\right|. \tag{15}$$

Note that $\sum_{\mathbf{k}}=\frac{1}{(2\pi)^2}A\int d^2\mathbf{k}=\frac{mA}{2\pi}\int\frac{k}{m}dk$, where A is the area of the system. We use the replacement $\frac{k}{\sqrt{m}}\rightarrow k$, thus we obtain

$$\frac{2\pi\mathcal{E}}{mA} = 2\Delta^2\int kdk\frac{1}{k^2+\epsilon_b} + \int k^3dk - \int 2\mu kdk - \sum_{\pm}\int kdk\left|\sqrt{\left(\frac{k^2}{2}-\mu\right)^2+\Delta^2}\pm\Omega\right|. \tag{16}$$

We will set a cutoff momentum λ for the integral, and then expand the result by $1/\lambda$. Finally we let the cutoff λ to be infinite. For the first term in the Eq.(16), we have

$$\begin{aligned} 2\Delta^2\int_0^{\lambda}kdk\frac{1}{k^2+\epsilon_b} &= \Delta^2\ln\left[\lambda^2+\epsilon_b\right] - \Delta^2\ln\epsilon_b \\ &= \Delta^2\ln\lambda^2 - \Delta^2\ln\epsilon_b + \Delta^2\cdot O(\frac{\epsilon_b}{\lambda^2}). \end{aligned} \tag{17}$$

On the other hand, the second and third terms are

$$\int_0^\lambda k^3 dk = \frac{1}{4}\lambda^4 \tag{18}$$

and

$$\int_0^\lambda 2\mu k dk = \mu\lambda^2 \tag{19}$$

respectively.

For the last term in Eq.(16), we consider two different cases. For $\Delta > \Omega$, we have $\sqrt{\left(\frac{k^2}{2}-\mu\right)^2+\Delta^2} \pm \Omega > 0$. In that case the integral becomes

$$\begin{aligned} a_4 &\equiv \sum_{\pm}\int_0^\lambda kdk \left|\sqrt{\left(\frac{k^2}{2}-\mu\right)^2+\Delta^2} \pm \Omega\right| \\ &= \frac{1}{2}\int_0^{\lambda^2} dk^2 \sqrt{\left(k^2-2\mu\right)^2+4\Delta^2} \\ &= \frac{1}{2}\int_{-2\mu}^{\lambda^2-2\mu} dx\sqrt{x^2+4\Delta^2}. \end{aligned} \tag{20}$$

By using

$$\int dx\sqrt{x^2+y^2} = \frac{1}{2}x\sqrt{x^2+y^2} + \frac{1}{2}y^2 \ln\left|\frac{\sqrt{x^2+y^2}+x}{y}\right| + Const. \tag{21}$$

we have

$$\begin{aligned} a_4 &= \left[\frac{1}{4}\left(\lambda^2-2\mu\right)^2 + \frac{\Delta^2}{2} + \Delta^2 O\left(\frac{\Delta^2}{\lambda^4}\right)\right] + \Delta^2\left[\ln\frac{\lambda^2-2\mu}{2\Delta^2} + O\left(\frac{\Delta^2}{\lambda^4}\right)\right] \\ &\quad + \mu\sqrt{\mu^2+\Delta^2} - \Delta^2 \ln\frac{\sqrt{\mu^2+\Delta^2}-\mu}{2\Delta^2}, \end{aligned} \tag{22}$$

As a result, for $\Delta > \Omega$ and $\lambda \to \infty$, we get

$$\frac{2\pi\mathcal{E}}{mA} = \Delta^2 \ln\frac{\sqrt{\mu^2+\Delta^2}-\mu}{\epsilon_b} - \frac{\Delta^2}{2} - \mu\sqrt{\mu^2+\Delta^2} - \mu^2 \equiv f\left(\Delta, \epsilon_b, \mu\right). \tag{23}$$

For $\Delta < \Omega$, one has

$$\begin{aligned} &-\sum_{\pm}\int kdk \left|\sqrt{\left(\frac{k^2}{2}-\mu\right)^2+\Delta^2} \pm \Omega\right| \\ &= -2\int kdk\sqrt{\left(\frac{k^2}{2}-\mu\right)^2+\Delta^2} + g\left(\Delta, \Omega, \mu\right), \end{aligned} \tag{24}$$

where

$$g\left(\Delta, \Omega, \mu\right) = 2\int_S kdk[\sqrt{\left(k^2/2-\mu\right)^2+\Delta^2} - \Omega] \tag{25}$$

and S is the region that $\sqrt{\left(\frac{k^2}{2}-\mu\right)^2+\Delta^2} < \Omega$, i.e., $\mu - \sqrt{\Omega^2-\Delta^2} < \frac{k^2}{2} < \mu + \sqrt{\Omega^2-\Delta^2}$. In the following, we suppose that $\mu - \sqrt{\Omega^2-\Delta^2} > 0$ and try to calculate out the integral $g\left(\Delta, \Omega, \mu\right)$. (Note that if $\mu + \sqrt{\Omega^2-\Delta^2} < 0$,

then the region S vanishes.) Performing the integration, one has

$$2\int_S kdk\sqrt{\left(\frac{k^2}{2}-\mu\right)^2+\Delta^2}=2\Omega\sqrt{\Omega^2-\Delta^2}+\Delta^2\ln\frac{\Omega+\sqrt{\Omega^2-\Delta^2}}{\Omega-\sqrt{\Omega^2-\Delta^2}}, \tag{26}$$

and

$$-2\int_S kdk\Omega=-\int_{2\left[\mu-\sqrt{\Omega^2-\Delta^2}\right]}^{2\left[\mu+\sqrt{\Omega^2-\Delta^2}\right]}dk^2\Omega=-4\Omega\sqrt{\Omega^2-\Delta^2}. \tag{27}$$

Note that these two integrals are independent on μ, so one can write $g\left(\Delta,\Omega\right)\equiv g\left(\Delta,\Omega,\mu\right)$. Finally, we have

$$\frac{2\pi\mathcal{E}}{mA}=f\left(\Delta,\epsilon_b,\mu\right)+\Theta\left(\Omega-\Delta\right)g\left(\Delta,\Omega\right), \tag{28}$$

where $g\left(\Delta,\Omega\right)=\Delta^2\ln\frac{\Omega+\sqrt{\Omega^2-\Delta^2}}{\Omega-\sqrt{\Omega^2-\Delta^2}}-2\Omega\sqrt{\Omega^2-\Delta^2}$.

B. Ground energy for a zero Raman coupling limit

In the limit of zero Raman coupling limit, one has $E_{\alpha,\mathbf{k}}^{\Omega=0}\equiv\sqrt{\left(\frac{k^2}{2m}-\mu\pm t\right)^2+\Delta^2}$, where we have used the fact $\Delta_{\mathrm{I}}=0$. It has two effective chemical potentials $\mu_\pm=\mu\pm t$. Using the result in the last subsection, it is easy to obtain the ground energy

$$\frac{2\pi\mathcal{E}^{\Omega=0}}{mA}=\frac{1}{2}f\left(\Delta,\epsilon_b,\mu+t\right)+\frac{1}{2}f\left(\Delta,\epsilon_b,\mu-t\right). \tag{29}$$

C. Ground energy Raman coupling with $\varphi=0$

For $\varphi=0$, we have $E_{\alpha,\mathbf{k}}^{0}=\left|\sqrt{\left(\frac{k^2}{2m}-\mu_\pm\right)^2+\Delta^2}\pm\Omega\right|$, where $\mu_\pm=\mu\pm t$ and we have used the fact $\Delta_{\mathrm{I}}=0$. Similar to the previous cases, for $\Delta>\Omega$, we have ground energy

$$\frac{2\pi\mathcal{E}}{mA}=\frac{2\pi\mathcal{E}^{\Omega=0}}{mA}. \tag{30}$$

On the other hand, for $\Delta<\Omega$ and $\mu_\pm-\sqrt{\Omega^2-\Delta^2}>0$, using the method in Eqs. (24), (26) and (27), we have

$$\frac{2\pi}{mA}\mathcal{E}=\frac{2\pi}{mA}\mathcal{E}^{\Omega=0}+\Theta(\Omega-\Delta)g\left(\Delta,\Omega\right). \tag{31}$$

For another case, $\Delta<\Omega$, $\mu_-+\sqrt{\Omega^2-\Delta^2}<0$ and $\mu_+-\sqrt{\Omega^2-\Delta^2}>0$, where the integral region S vanishes (See Eq.(25)) for the μ_- branch, the ground energy becomes

$$\frac{2\pi}{mA}\mathcal{E}=\frac{2\pi}{mA}\mathcal{E}^{\Omega=0}+\frac{1}{2}\Theta(\Omega-\Delta)g\left(\Delta,\Omega\right). \tag{32}$$
